# Hepatitis B Vaccines

**DOI:** 10.1093/infdis/jiaa668

**Published:** 2021-09-30

**Authors:** Jade Pattyn, Greet Hendrickx, Alex Vorsters, Pierre Van Damme

**Affiliations:** Centre for the Evaluation of Vaccination, University of Antwerp, Antwerp, Belgium

**Keywords:** hepatitis B virus, hepatitis B, hepatitis B vaccination

## Abstract

Hepatitis B is caused by the hepatitis B virus (HBV), which infects the liver and may lead to chronic liver disease, including cirrhosis and hepatocellular carcinoma. HBV represents a worldwide public health problem, causing major morbidity and mortality. Affordable, safe, and effective, hepatitis B vaccines are the best tools we have to control and prevent hepatitis B. In 2019, coverage of 3 doses of the hepatitis B vaccine reached 85% worldwide compared to around 30% in 2000. The effective implementation of hepatitis B vaccination programs has resulted in a substantial decrease in the HBV carrier rate and hepatitis B-related morbidity and mortality. This article summarizes the great triumphs of the hepatitis B vaccine, the first anticancer and virus-like-particle–based vaccine. In addition, existing unresolved issues and future perspectives on hepatitis B vaccination required for global prevention of HBV infection are discussed.

## HEPATITIS B

### What is Hepatitis B?

Viral hepatitis type B caused by the hepatitis B virus (HBV) is a serious, potentially life-threatening disease that can be prevented by vaccination. Most people that are (newly) HBV infected remain asymptomatic and do not know their hepatitis status for many years. Only some individuals newly infected with HBV have symptoms (acute hepatitis) [1, 2]. The symptoms can include extreme fatigue, abdominal pain, nausea, and jaundice. Most available scientific evidence suggests that HBV is not directly cytopathic, but that liver damage is caused by the cellular response to viral proteins in infected hepatocytes [3]. For many people, hepatitis B is a short-term illness as clinical signs and symptoms of acute hepatitis B usually resolve within 1 to 3 months [4]. Fulminant liver failure occurs in approximately 0.5% to 1.0% of adults with reported acute hepatitis B. In a subset of persons, the HBV can also cause a chronic liver infection that can later develop into cirrhosis (scarring of the liver) or hepatocellular carcinoma (HCC). The course of chronic HBV infection is dynamic with different clinical phases, each of which potentially lasts for decades [5, 6]. Most of the disease burden associated with HBV infection occurs among persons with chronic infection [7].

The age of acquisition of the HBV infection is the main determining factor in the clinical expression of acute disease and the development of chronic infection [8, 9]. Fewer than 10% of children younger than 5 years who become infected have initial clinical signs or symptoms of disease (ie, acute hepatitis B) compared to 30% of infections in adults [10, 11]. The risk of developing chronic HBV infection varies inversely with age; 80%–90% of infants infected during their first year of life develop chronic infections, as opposed to 30%–50% of children infected before the age of 6 years and 1%–5% of adults [10, 11]. Neonatal immune tolerance to viral antigens appears to play an important role in viral persistence in infants infected at birth [12, 13]. Because HBV infections occurring perinatally, during infancy, or in early childhood are most likely to become chronic, vaccination of new-borns and infants is today a key intervention for prevention of HBV infections.

### What is the Morphology of HBV?

**Figure 1.**
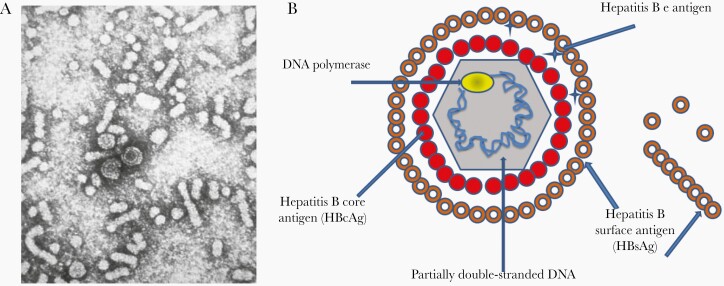
*A*, Electron micrograph of hepatitis B virus (HBV): Dane particles (43 nm) and spherical and tubular surface antigen particles (22 nm) [4]. Source: Centers for Disease Control and Prevention. As a work of the U.S. federal government. *B*, A simplified figure of the HBV particle and surface antigens.

HBV is an oncogenic DNA virus that belongs to the Hepadnaviridae family. The discovery of the etiologic agent of hepatitis B remains a remarkable scientific achievement. It was discovered in 1965 by Dr Blumberg, who won the Nobel Prize in Medicine for his discovery in 1976 [14]. HBV virus, initially called the Dane particle, is a 42-nm virus [15]. HBV is composed of a nucleocapsid core, surrounded by an outer lipoprotein coat (also called envelope). The virus contains 3 primary structural antigens: surface (HBsAg), core (HBcAg), and e (HBeAg) (Figure 1) [4]. HBsAg is produced in excess amounts and found in the blood of infected individuals in the form of spherical and tubular particles (approximately 22 nm). These immunogenic, but noninfectious, subviral particles lack genomic DNA and paved the way to develop hepatitis B vaccines [16–18]. HBV is divided into 4 major phenotypic subtypes (adw, adr, ayw, and ayr) based on antigenic epitopes presented on its envelope proteins, and comprises 10 major genotypes (A to J) that differ at the nucleotide level across full-length genotypes by > 8% [19]. The HBV genotypes have distinct virological characteristics and geographical distributions [20]; however, the licensed HBV vaccines are effective against all genotypes [21, 22].

### What is the Transmission Route of HBV?

Today, we know that HBVs are highly infectious and are spread by exposure of mucosal membranes or nonintact skin to infected blood or other body fluids (saliva, semen, and vaginal fluid) [4]. In high endemic areas, HBV is most commonly transmitted from mother to child at birth (perinatal transmission) and during early childhood from infected to uninfected children (horizontal transmission). Most HBV infections in areas of low endemicity occur in adults in relatively well-defined risk groups, such as those at risk through sexual exposure, household members of an infected person, hemodialysis patients, incarcerated persons, injection-drug users, persons at risk for occupational exposure, developmentally disabled persons in long-term care facilities, and travelers to regions with moderate or high HBV endemicity. Today, only humans are a known reservoir for human HBV genotypes, but closely related HBV genotypes exist in higher primates [23]. Hence, a comprehensive control strategy could eventually lead to the eradication of HBV.

### How is Hepatitis B Diagnosed?

Because clinical manifestations of hepatitis B are indistinguishable from other causes of viral hepatitis, a definitive diagnosis requires serological testing [24]. This testing uses different (combinations of) serologic markers to identify different phases of HBV infection and to conclude whether a person has acute or chronic HBV infection, or is immune to HBV as a result of prior HBV infection or vaccination, or is susceptible to infection [4]. In Table 1 an interpretation of hepatitis B serologic test results is given.

**Table 1.** Interpretation of Serologic Test Results for Hepatitis B

	Acute HBV	Chronic HBV	Cleared HBV	Vaccination
HBcAb IgM	+	−	−	−
HBcAb IgG	+	+	+	−
HBsAg	+	+	−	−
Anti-HBs	−	−	+	+
HBeAg	+	+/−	−	−
Anti-HBe	−	+/−	+/−	−
HBV DNA	High/low	Low/high	−	−

Abbreviations: HBcAb IgG, hepatitis B core antibody immunoglobulin G; HBcAb IgM, hepatitis B core antibody immunoglobulin M; HBeAg, hepatitis B e antigen; HBsAg, hepatitis B surface antigen; HBV, hepatitis B virus.

Chronic infection is arbitrarily defined by the persistence of HBsAg (with or without concurrent HBeAg) in serum for at least 6 months. Anti-HBs are neutralizing antibodies and their presence in serum confers long-term immunity against HBV infection. In persons with acquired immunity through vaccination, anti-HBs is the only serological marker detected. In contrast to persons with a past HBV infection where anti-HBs are present concurrently with anti-HBc IgG.

During the initial, highly replicative phase of HBV infection patients are also seropositive for HBeAg, of whom some remain HBeAg positive for years. All HBsAg-positive persons should be considered infectious; however, the presence of HBeAg indicates that the blood and body fluids of the infected individual are highly contagious (ie, HBV DNA levels of 10^7^ to 10^9^ IU/mL). Although HBsAg has been detected in multiple body fluids, only serum, saliva, semen, and vaginal fluid have been demonstrated to be infectious [25].

### What Are the Treatment Options for Hepatitis B?

Currently, there is no specific antiviral treatment recommended for persons with acute hepatitis B disease as approximately 95% of infected immunocompetent adults recover spontaneously. Specific treatment is available to support people with chronic HBV infection. The main goal of the available care is to maintain comfort, relieve symptoms, and prevent patients from passing the infection to others. However, notably, not all patients with chronic HBV need to be on medication [2]. Patients with active signs of liver disease may benefit the most from current treatment. The Food and Drug Administration has approved for the treatment of chronic hepatitis B interferon-α (standard and pegylated) and oral antiviral agents (entecavir, tenofovir, dipovoxil fumarate, tenofovir alafenamide, and nonpreferred lamivudine, adefovir dipivoxil, and telbivudine). Present treatment for chronic hepatitis B can slow or prevent the progression of cirrhosis, reduce the incidence of liver cancer, and improve long term survival and quality of life, but are not curative. Therefore, most people who start hepatitis B treatment must continue for life. The side effects of the therapies and required regular monitoring increases the difficulty and complexity of patient management. Hence, hepatitis B vaccination is plan A in the fight against hepatitis B. Vaccination is, compared to other interventions, an economically attractive option, both in terms of cost-effectiveness and benefit-cost ratios [26].

### The Burden of HBV

**Figure 2.**
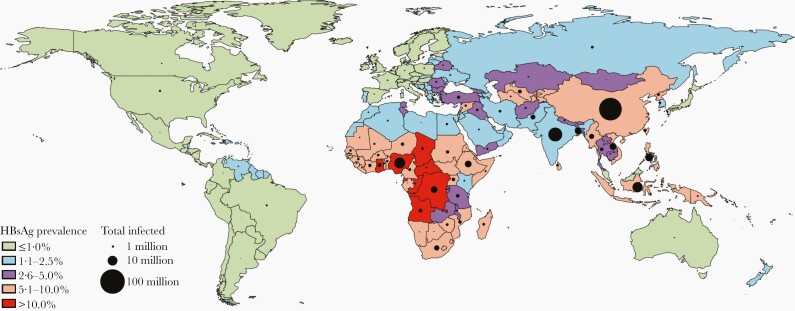
Hepatitis B surface antigen (HBsAg) prevalence estimates for 2016. Estimates for countries with data and a model or for which data were extrapolated from countries in the same Global Burden of Disease region with available data (all ages). From Polaris Observatory Collaborators. Global prevalence, treatment, and prevention of hepatitis B virus infection in 2016: a modelling study [27].

Hepatitis B immunization is very important as HBV infection is still the leading cause of liver cancer and causes significant morbidity and mortality worldwide. In 2015, an estimated 887 220 persons died as a result of HBV infection globally (87 076 due to acute hepatitis, 462 690 due to cirrhosis, and 337 454 due to HCC) [1]. Furthermore, the World Health Organization (WHO) estimates that, in 2015, 257 million people were living with a chronic HBV infection, the frequency and burden of which vary by region and subpopulation (Figure 2). As of 2016, 27 million people (10.5% of all people estimated to be living with hepatitis B) were aware of their infection, while only 4.5 million (16.7%) of the people diagnosed were on treatment [1]. However, due to successful vaccination programs, the epidemiology of HBV has been changing over the recent years [28].

## THE SUCCESS OF HEPATITIS B IMMUNIZATION

### History and Development of the First Anticancer Vaccine

The first hepatitis B vaccines, commercially available since 1982, were plasma-derived vaccines. The American microbiologist Maurice Hilleman produced these vaccines by harvesting subvirion particles of HBsAg (22-nm particles) from the plasma of asymptomatic chronic HBV-infected donors [16]. The particles in the pooled plasma were highly purified and any residual infectious particles were inactivated by various combinations of urea, pepsin, formaldehyde, and heat [17]. Plasma-derived vaccines have been investigated with success in several hundred million individuals, leading to the first licensed hepatitis B vaccines. This first HBV vaccines were manufactured under the name Heptavax B (Merck) and Hevac B (Institut Pasteur) and targeted a number of high-risk groups, the main focus of the immunization program at that time. Although concerns about the safety of these vaccines regarding transmission of bloodborne pathogens, including human immunodeficiency virus (HIV), have proven to be unfounded, public anxieties over the safety of the plasma-derived vaccine persisted and hampered its acceptance in many populations [29]. Other barriers for high coverage included high vaccine costs and the lack of global vaccine policies [18].

In 1986, the first genetically engineered hepatitis B vaccine was developed with recombinant HBsAg, resulting in a second-generation of HBV vaccines that completely replaced plasma-derived vaccines [30–32]. The development of recombinant DNA technology to express HBsAg in yeast, and later also in mammalian cells, offered the potential to produce large quantities of vaccine [19]. The yeast-derived recombinant vaccines, which are the most widely used, are manufactured by expression of HBsAg protein in genetically engineered yeast cells (*Saccharomyces cerevisiae*) that contain the S gene. HBsAg of HBV self-assembles into virus-like particles (VLPs) and its use as a vaccine results in protective antiviral immunity against HBV infections. The recombinant hepatitis B vaccines are a memorable milestone in the field of vaccinology as they were the first vaccines based on VLPs.

The recombinant hepatitis B vaccines are highly immunogenic. A variety of immunization schedules have been shown to induce levels of seroprotection of greater than 95% in healthy infants, children, and young adults [4]. However, studies indicated that older adults (>40 years of age) are less likely to achieve a seroprotective response to hepatitis B vaccination and this drops to 60%–70% in adults aged 60 years and older [33]. Obesity, smoking, HIV infection, genetic factors, and chronic disease may also result in lower response rates. The recombinant HBsAg particles differ from natural viral particles in lacking the preS domain of HBsAg and lacking glycosylation due to their production in yeast [34]. Mammalian cell-derived vaccines contain glycosylated pre-S1 and pre-S2 proteins, in addition to the major HBsAg protein. By covering not only the HBsAg S epitope, these vaccines, as well as some new vaccine adjuvants formulations that have been and are being developed, have been shown to be more immunogenic compared to the second-generation vaccines. Although they are too costly to be included in the universal vaccination programs, they can confer protection to immunocompromised persons and nonresponders [35].

### Implementing Strategies for Hepatitis B Vaccination

When hepatitis B vaccines became available, strategies for HBV control were initially focused on vaccination of high-risk groups [28]. However, high-risk individuals are mostly difficult to reach and are often infected before vaccination [36]. Consequently, coverage of 3 doses of hepatitis B vaccine remained low in most high-risk groups due to low compliance and logistic reasons [4]. In addition, as many as 30% or more people with acute hepatitis B infection do not have identifiable risk factors and are therefore missed by only a high-risk group approach [2].

Hence it was clear that an additional global strategy was required as the high-risk strategy made little impact and the global burden of hepatitis B diseases became more and more obvious. Decision makers and health professionals worldwide started to discuss a strategy of universal hepatitis B immunization for a certain age cohort, even in low-endemicity countries. In 1991, the WHO’s Global Advisory Group of the Expanded Programme on Immunization recommended that hepatitis B vaccine be integrated into national immunization programs in all countries by 1997 [37]. This 1991 recommendation was endorsed by the World Health Assembly in 1992 [38]. Progressively, it has become more widely used and recommendations for HBV vaccination have been extended in an attempt to achieve maximum protection [39].

Because perinatal and early postnatal transmission are the major cause of chronic hepatitis infection, hepatitis B vaccination is now recommended by the WHO for all infants beginning at birth (universal vaccination) and children and adolescents who did not receive the hepatitis B vaccine during infancy (catch-up vaccination). For adults, it is still recommended for those who are at increased risk for HBV infection [19]. The need for immunizing these high-risk people is determined by the baseline epidemiology of HBV infection in the country. Generally, implementation of routine infant immunization produces broad population-based immunity to HBV infection and eventually prevents HBV transmission in all age groups. Hence, the catch-up vaccination is now prioritized for younger age groups because the risk of chronic infection is the highest in these cohorts [2].

To provide maximal protection against mother-to-child transmission, the first dose of (monovalent) hepatitis B vaccine should be given as soon as possible (<24 hours) after birth [40]. In randomized, placebo-controlled trials, administration of hepatitis B vaccine in a 3- or 4-dose schedule, beginning less than 12 hours after birth prevents 70% to 95% of perinatal HBV infections among infants born to highly infectious women who are positive for both HBsAg and HBeAg [41]. The effectiveness of the birth dose in preventing perinatal transmission declines progressively in the days after birth; however, a late birth dose may still be partially effective [40, 42]. As of 2016, 101 (52%) countries had introduced this WHO-recommended birth dose [43]. However, in some settings, the administration of a birth dose is restricted by the high proportions of births in nonmedical facilities, lack of trained health care staff, poor political commitment, and logistical and cultural issues [44]. A substantial burden of chronic HBV infection persists because the global coverage of the timely birth dose is still low, estimated globally at 42% by the end of 2018 [43]. In 20 (10%) countries, where a program of screening for all pregnant women has been installed, the hepatitis B birth dose was introduced only for infants born to HBsAg-positive mothers. In low- and middle-income countries as well as in industrialized countries screening of all pregnant women before delivery, offering timely neonatal vaccination, and monitoring HBsAg and anti-HBs in vaccinated infants from HBsAg positive mothers remains a clear challenge [45].

### HBV Vaccination Doses and Formulations

Given differences in the manufacturing processes and populations vaccinated, the quantity of HBsAg protein per dose that will induce a protective immune response differs in various vaccine products. Currently, hepatitis B vaccines are formulated to contain 5–40 μg of recombinant HBsAg protein and an aluminum phosphate or aluminum hydroxide adjuvant [4]. In general, based on immunogenicity data with different vaccine dosages in different age groups, the vaccine dosages to provide protection for infants, children, and adolescents are 50% lower than that required for adults [46, 47]. Marketed hepatitis B vaccines are to be administered by intramuscular injection on the anterolateral site of the thigh (for infants and children aged < 2 years) or into the deltoid muscle (for older children and adults). The WHO has developed recommendations to ensure the quality, safety, and efficacy of recombinant hepatitis B vaccines and keeps a list of current hepatitis B vaccines prequalified by the WHO [48].

Hepatitis B vaccines are available as monovalent formulations for birth doses or for vaccination of adult persons at risk, and as combination vaccines (eg, infant vaccination, including diphtheria-tetanus-pertussis, *Haemophilus influenzae* type b, and inactivated polio vaccine). Major progress in the global response to viral hepatitis has been achieved through the introduction of routine hepatitis B vaccination via the WHO’s Expanded Programme on Immunization, which was facilitated by the introduction of combination vaccines [2]. Hepatitis B vaccines are generally stable for 3 to 4 years from the date of manufacture if stored between 2°C and 8°C.

### The Impact of Worldwide Hepatitis B Vaccination Programs: Model of Success

**Figure 3.**
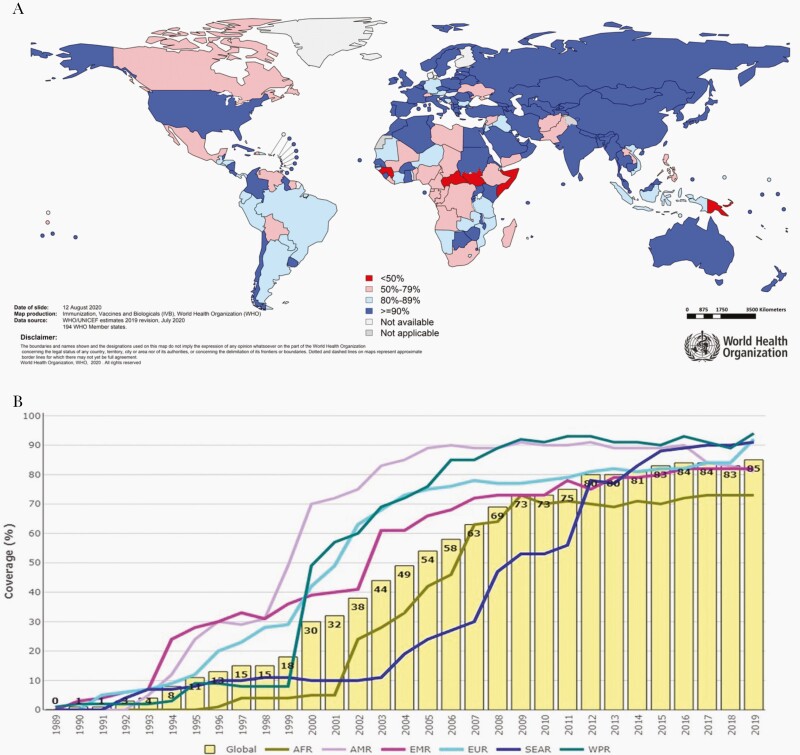
*A*, Immunization coverage with third dose of hepatitis B (HepB3) in infants in 2019. *B*, Global immunization 1989–2019 HepB3 coverage in infants. Global coverage was 84% in 2019. Abbreviations: AFR, African region; AMR, Americas region; EMR, Eastern Mediterranean region; EUR, European region; SEAR, South-East Asia region; WPR, Western Pacific region. Source: United Nations Children's Fund (UNICEF)/World Health Organization.

By the end of 2019, 189 (97%) countries had incorporated hepatitis B vaccination in their national immunization schedule (Figure 3A). Globally, major progress has been achieved with immunization coverage with 3 doses of hepatitis B vaccine during infancy increasing from 1% in 1990 to 85% in 2019 (Figure 3B) [43]. This achievement was made possible through several developments, including removal of cost-related barriers (<$1 per dose) and because of the financial and logistical support from the Global Alliance for Vaccination and Immunization (GAVI) that has allow the world’s poorest countries to obtain the vaccine since 2001. Pentavalent vaccine coverage in GAVI-supported countries increased from 1% in 2000 to 81% in 2019. However, there are still substantial variations across WHO regions. The Western Pacific region (90%), the American region (89%) and the South East Asia regions (87%) are currently above the global average, while the European region (81%), the Eastern Mediterranean region (80%), and the African region (75%) have lower coverage [1].

The success of HBV vaccination programs has been clearly demonstrated over the recent years in several regions around the world. Countries that have adopted the recommendation had a marked reduction in carrier rates as well as complications from HBV, including HCC. The low prevalence of chronic HBV infection in children younger than 5 years, reducing from 4.7% in the prevaccine era to less than 1% in 2019, can be attributed to the widespread use of hepatitis B vaccine. Due to the implementation of routinely birth-dose vaccination the greatest decrease appears to be in the Western Pacific region, from 8.3% HBsAg prevalence in the prevaccine era to 0.93% in 2002–2015 [49]. Among health care workers, hepatitis B vaccination is highly effective for the prevention of healthcare associated HBV infection and chronic infection. Using mathematical models, it was estimated that since their implementation, HBV vaccination programs have averted 210 million new HBV infections globally [50].

Countries that were early to adopt and implement universal hepatitis B immunization include Taiwan (1984), Bulgaria (1989), Malaysia (1990), The Gambia (1990), Italy (1991), Spain (1991), the United States (1991), and Israel (1992). Taiwan is perhaps the best early success story of the hepatitis B vaccine in an area with previously high endemicity showing a substantial decrease in transmission and the burden of hepatitis B infection and HBV-related diseases after the introduction in 1984 of mass vaccination of new-borns. The HBsAg prevalence in individuals younger than 20 years decreased from 9.8% in 1984 to 1.3% in 1994, and to 0.6% in 2004, and has consistently remained < 1% [51]. The annual average incidence of HCC among children from 6 to 14 years old decreased from 0.7 per 100 000 in 1981 through 1986, to 0.36 per 100 000 in 1990 through 1994. In 2004, the HCC incidence for age groups of 6 to 9, 10 to 14, and 15 to 19 years decreased to 0.15, 0.19, and 0.16 per 100 000 person-years, respectively, clearly indicating the hepatitis B vaccine to be the first successful vaccine against cancer.

### Why a Variety of Hepatitis B Vaccine Schedules Have Been Used

Generally, the recommended number of doses of hepatitis B vaccine required to induce protective immunity varies by product and with the age of the recipient. Historically, the standard 3-dose hepatitis B vaccine series has consisted of 2 priming doses administered 1 month apart and a third dose administered 6 months after the first dose. Today, the WHO recommends multiple options for adding hepatitis B vaccine to existing infant immunization schedules. Several options are considered to be appropriate for infants: 1 (monovalent) birth dose followed by either 2 doses of monovalent or hepatitis B containing combination vaccine at 1 and 6 months of age; or at 2, 4, and 6 months of age; or at 3, 5, and 11 months of age; or at 8, 12, 16 weeks and 12 or 15 months; or at 6, 10, and 14 weeks of age, according to the WHO’s Expanded Programme on Immunization schedule [4]. Currently, a variety of hepatitis B vaccine schedules have been used successfully worldwide. In general, preference is given to effective options that require minimal additional visits for immunization, to increase compliance and to reduce the logistics burden.

IgG antibodies to HBsAg (anti-HBs) after completion of vaccination are used as a marker of immunity to HBV. An anti-HBs antibody concentration of 10 mIU/mL or more measured 1–3 months after the administration of the last dose of the primary vaccination series is considered a reliable marker of protection against HBV infection [4, 52]. This correlate of protection was determined by Jack and colleagues who followed children vaccinated with the hepatitis B vaccine in the Gambia, and correlated their antibody levels with subsequent incidence of HBsAg carriage [53]. The results suggested that reaching a peak antibody level of > 10 mIU/mL in the year following vaccination was associated with protection against hepatitis B persistent infection over the subsequent 7 years [53].

### The Safety of Hepatitis B Vaccination Programs

Numerous clinical trials and widespread practical applications have demonstrated that hepatitis B vaccines are very safe. Since 1982, over 1 billion doses of hepatitis B vaccine have been used worldwide. Adverse events after immunization against hepatitis B are infrequent and generally mild and transient. Except for localized pain, placebo-controlled studies have revealed that reported events (eg, myalgia and transient fever) occur no more frequently among vaccinees than among persons receiving placebo [1]. Data from numerous long-term studies fail to causally link other serious adverse events to hepatitis B vaccination. Data do not indicate a causal association between hepatitis B vaccine and neurological disease (including Guillain-Barré syndrome and multiple sclerosis), leukemia, diabetes mellitus, demyelinating disorders, chronic fatigue syndrome, arthritis, autoimmune disorders, asthma, hair loss, or sudden infant death syndrome. The Global Advisory Committee on Vaccine Safety has confirmed the excellent safety profile of hepatitis B vaccine and continues to monitor the safety of this vaccine. Furthermore, hepatitis B vaccination can be administered safely to pregnant women during any trimester of pregnancy and to breastfeeding women. Both low birth weight and premature infants and HIV-positive persons can receive hepatitis B vaccination. Hepatitis B vaccination is contraindicated only for persons with a history of allergic reactions to yeast or any of the vaccine’s components.

### The Duration of Protection and the Need for Booster Doses

In vaccine efficacy studies, immunocompetent children and adults who developed anti-HBs concentrations of 10 mIU/mL or higher after vaccination had complete protection against both acute disease and chronic infection for decades (documented for up to 30 years so far), even if subsequently, over time, anti-HBs concentrations declined to less than 10 mIU/mL [54, 55]. Indeed, the protective efficacy of hepatitis B vaccination is related to the induction of anti-HBs antibodies, but it also involves the induction of memory B and T cells. Ongoing surveillance of vaccinees is required to clarify whether hepatitis B vaccination can confer longer, or even lifelong, protection [56]. Based on currently available scientific evidence, different advisory groups do not recommend routine booster doses of hepatitis B vaccine in immunologically competent persons who have received a full primary course, because the majority of previously vaccinated people with an anti-HBs antibody concentration of 10 mIU/mL or less mount an anamnestic response when they receive a booster dose or are exposed to HBV, indicating that they remained protected by memory B and T cells [57].

### The Cost-Effectiveness of Hepatitis B Vaccination Programs

HBV infection causes worldwide a high burden in terms of costs, both direct and indirect. Since the introduction of the hepatitis B vaccine, scientific evidence suggests hepatitis B vaccination is one of the most cost-effective public health interventions available [58, 59]. Likewise, birth-dose hepatitis B vaccination has been shown to be cost-effective regardless of HBV endemicity. Key drivers influencing cost-effectiveness are the prevalence of HBsAg and vaccine-related factors such as vaccine price, discount rate, wastage rate of vaccine, and vaccine efficacy [60]. Besides the health and economic benefits of vaccination to society, the social benefits are to be considered in the overall assessment of impact (eg, equity of health care, strengthening health and social care infrastructure, impact on life expectancy and opportunity, and empowerment of women) [61, 62].

## CHALLENGES AND FUTURE CONSIDERATIONS

### WHO Strategy for Hepatitis B Immunization

Although major progress has been achieved in hepatitis B immunization, a number of challenges remain. That is why the WHO called for comprehensive prevention and control of HBV infection and the development of time-specific immunization goals in its member states. The strategy includes the following: (1) universal vaccination of infants within 24 hours of birth, (2) full immunization of infants by routine immunization programs, (3) catch-up vaccination of unimmunized cohorts, and (4) monitoring progress and assessing the impact of immunization [2].

#### Universal Vaccination of Infants Within 24 Hours of Birth: a Real Challenge

In several regions, the hepatitis B birth-dose coverage remains low as a result of a combination of setting-dependent determinants. In low-resource settings particularly, vaccine availability, storage, and transportation in an appropriate cold chain, as well as high rates of births outside health facilities, are hurdles. In addition, other barriers to overcome stem from lack of understanding by parents, healthcare providers, or policymakers of the extensive safety record and benefits of administering hepatitis B vaccine at birth. Health promotion efforts are needed to eliminate false contraindications, to allay concerns over the theoretical risk of adverse reactions, fear of vaccine wastage, personal cost concerns, and cultural prohibitions. If well executed, hepatitis B birth dose could interrupt the majority of vertical transmission within a generation, thereby closing the gap in immunity between birth and primary series. By the beginning of 2021, GAVI has agreed to provide support to establish (new) platforms as catalytic support for the introduction of hepatitis B administered at birth.

#### Full Immunization of Infants by Routine Immunization Programs and Catch-Up Vaccination of Unimmunized Cohorts

Wider provision of the existing, safe and effective HBV vaccine, through universal childhood vaccination and by catch-up vaccination of unimmunized cohorts, will further reduce new hepatitis B infections, reducing rates of chronic illness and death. However, to achieve and/or sustain high coverage, stronger and resilient immunization delivery systems will be needed. Still, some countries (eg, Denmark, Finland, Iceland, and Sweden) adopt risk-group–targeted vaccination only, instead of adding a universal vaccination program. However, changing demography, increasing immigration, and the current vaccine costs make the cost-benefit ratios in these remaining low-endemicity countries strongly in favor of universal HBV vaccination.

#### Monitoring Progress and Assessing the Impact of Immunization

For hepatitis B, further surveillance is needed to generate the long-term impact of the immunization and control programs. Serological surveys of HBsAg prevalence, representative of the target population, will serve as the primary tool to further measure the impact of vaccination and verify the achievement of the hepatitis B control goals. To ensure sustained support, the success associated with immunization should be measured, and also communicated.

### Delivering Hepatitis B Vaccines Outside the Cold Chain

Traditionally, hepatitis B vaccine should be transported and stored at 2–8°C, so-called cold chains. Freezing must be avoided as it causes dissociation of the antigen from the alum adjuvant, resulting in loss of potency. Nevertheless, a WHO review on in vivo and in vitro testing to assess the thermostability of monovalent hepatitis B vaccines, suggests that hepatitis B vaccines are relatively heat stable [63–65]. Hence, WHO recommends vaccines to be licensed for use in a controlled temperature chain (CTC). A CTC is a specifically defined approach to vaccine delivery allowing vaccines to be kept at temperatures beyond the traditional cold chain of 2–8°C for a limited period of time, under monitored and controlled conditions. A CTC typically involves a single excursion of the vaccine into ambient temperatures not exceeding 40°C and for a specific number of days, immediately prior to administration. The vaccines should be accompanied by a vaccine vial monitor on each vial, and a peak threshold indicator in each vaccine carrier. Storage of hepatis B vaccines outside the cold chain could help to increase the birth dose where access to health care is geographically challenging, home births are common, or health facilities lack adequate cold chain capacity. Although CTC has the potential to save many lives and money, few manufacturers have been willing to commit to the additional stability testing and label variation needed. Consequently, global efforts to support manufacturers are needed as HBV vaccination programs would be greatly enhanced by the use of a more heat-stable and freeze-stable vaccine and simplified cost-saving delivery systems [66].

### The Nonresponder Situation as a Challenge

New vaccine formulations have been and are being developed to meet the challenges of nonresponse or low response among older adults and immunocompromised individuals to current hepatitis B vaccines, for example, third-generation hepatitis B recombinant vaccines containing HBsAg, preS1, and pre-S2 antigens [35] or adjuvanted hepatitis B recombinant vaccines (eg, HBsAg-1018 ISS [67, 68]). These vaccines are showing improved immune response in immunocompromised populations and older adults and, in addition, these new vaccines can offer the possibility of simplified schedules, which might be very promising for the future, for example, a 0, 1-month schedule instead of the traditional 0, 1, 6-month schedule [69, 70].

### Duration of Protection

The duration of protection after hepatitis B vaccination is not exactly known yet. Among children who respond to a primary 3-dose vaccination series with anti-HBs concentrations of 10 mIU/mL or greater, 15% to 50% have low or undetectable concentrations of anti-HBs 5 to 15 years after vaccination [4]. However, protection has been shown to outlast the presence of vaccine-induced antibodies, conferring effective long-term protection against acute disease and development of HBsAg carriage for up to 20 to 30 years now [71]. These results explain the current recommendations that a booster is not needed in healthy, fully vaccinated, immunocompetent adults. Nevertheless, additional long-term follow up studies are needed to explore longer or even life-long protection conferred by hepatitis B vaccine; moreover, the need for a booster after a number of years should also be evaluated. Besides, it is still a concern whether the long-term protection in vaccinees who received a reduced quantity of HBsAg (eg, 2.5 μg dosage) or in individuals with high-risk of HBV exposure is appropriately maintained [72, 73].

### Novel (Therapeutic) Hepatitis B Vaccines

As current treatment for chronic hepatitis B is not curative, therapeutic immunization for persons with chronic HBV infection and restoring the defective immune tolerance to HBV remains an important goal. Great efforts have been taken using different approaches to stimulate the humoral and/or cellular immune responses. However, until now, the clinical efficacy shown in clinical trials in humans is limited. Also, as indicated above, new vaccine formulations have been and are being developed to meet the challenges of nonresponse or low response among older adults and immunocompromised individuals. Additionally, novel injection devices (eg, Uniject) and intradermal, oral, and nasal prophylactic hepatitis B vaccines, which might be easier to administer than injections, are under investigation.

### Ending Hepatitis B as a Major Public Health Threat

Following the endorsement of the Global Health Sector Strategy on viral hepatitis (2016–2021) by the World Health Assembly, the WHO developed ambitious targets for the elimination of hepatitis, including HBV as public health threats by 2030 [74]. Service targets include further improvement in childhood vaccine coverage, major increases in birth-dose vaccine coverage or other methods for mother-to-child HBV prevention, and massive expansion of case finding were agreed on. These service improvements are aligned with impact targets of a 90% reduction in new HBV infections and a 65% reduction in HBV-related deaths (compared with 2015 WHO estimates). From the prevention perspective, global efforts are mostly on track for hepatitis B vaccination. The large scale-up of hepatitis B vaccine worldwide over the last 2 decades, which has been in large part due to the support provided by GAVI, has been a great public health success story and contributed to the decrease in HBV infections among children. However, expanding access to a timely birth dose of the hepatitis B vaccine will require major focused effort to increase coverage, as it is the cornerstone of efforts to prevent mother-to-children transmission of HBV. Although major progress has been made over the years, to achieve elimination (further) global and local political commitment will be essential, with the integration of prevention programs in the overall control and therapeutic plans. The battle against HBV is not over yet, but the broad use of hepatitis B vaccination is the cornerstone and most important tool we have to control HBV. We need to ensure financing, provision, distribution, and administration of vaccines to all populations, in particular those that are difficult to reach.

## Supplementary Data

Supplementary materials are available at *The Journal of Infectious Diseases* online. Consisting of data provided by the authors to benefit the reader, the posted materials are not copyedited and are the sole responsibility of the authors, so questions or comments should be addressed to the corresponding author.

The complete references are available as online Supplemental Material.

## Supplementary Material

jiaa668_suppl_Supplementary-Material

## Notes

***Supplement sponsorship.*** This supplement is sponsored by the Bill and Melinda Gates Foundation.

***Potential conflicts of interest.*** All authors: No reported conflicts of interest. All authors have submitted the ICMJE Form for Disclosure of Potential Conflicts of Interest. Conflicts that the editors consider relevant to the content of the manuscript have been disclosed.
