# Supplementary material for: Hepatitis B Vaccines
Source: J Infect Dis. 2021 Sep 30;224(Suppl 4):S343–51. doi: 10.1093/infdis/jiaa668 (PMC8482019; doi:10.1093/infdis/jiaa668)
Supplement: jiaa668_suppl_Supplementary-Material [file jiaa668_suppl_supplementary-material.docx]

**Supplementary Data**

1. World Health Organization (WHO). Fact sheet: hepatitis B. Geneva, Switzerland: WHO, 2019.
2. World Health Organization (WHO). Hepatitis B vaccines: WHO position paper. Geneva, Switzerland: WHO, 2017.
3. Chisari FV, Isogawa M, Wieland SF. Pathogenesis of hepatitis B virus infection. Pathol Biol (Paris) 2010; 58:258–66.
4. Van Damme P, Ward JW, Shouval D, Zanetti A. Hepatitis B Vaccines. In: Plotkin SA, Orenstein W, Offit PA, Edwards KM, eds. Plotkin’s vaccines. 7th Edn. Philadelphia, PA: Elsevier, 2017.
5. Carneiro de Moura M, Marinho R. Natural history and clinical manifestations of chronic hepatitis B virus [Spanish]. Enferm Infecc Microbiol Clin 2008; 26 (suppl 7):11–18.
6. Trépo C, Chan HL, Lok A. Hepatitis B virus infection. Lancet 2014; 384:2053–63.
7. Hoofnagle JH. Chronic hepatitis B. N Engl J Med 1990; 323:337–9.
8. Edmunds WJ, Medley GF, Nokes DJ, Hall AJ, Whittle HC. The influence of age on the development of the hepatitis B carrier state. Proc Biol Sci 1993; 253:197–201.
9. Shimakawa Y, Lemoine M, Bottomley C, et al. Birth order and risk of hepatocellular carcinoma in chronic carriers of hepatitis B virus: a case-control study in The Gambia. Liver Int 2015; 35:2318–26.
10. Wright TL, Lau JY. Clinical aspects of hepatitis B virus infection. Lancet 1993; 342:1340–4.
11. McMahon BJ. The natural history of chronic hepatitis B virus infection. Hepatology 2009; 49:S45–55.
12. Milich DR, Jones JE, Hughes JL, Price J, Raney AK, McLachlan A. Is a function of the secreted hepatitis B e antigen to induce immunologic tolerance in utero? Proc Natl Acad Sci U S A 1990; 87:6599–603.
13. Bauer T, Sprinzl M, Protzer U. Immune control of hepatitis B virus. Dig Dis 2011; 29:423–33.
14. Blumberg BS, Alter HJ, Visnich S. A “new” antigen in leukemia sera. JAMA 1965; 191:541–6.
15. Dane DS, Cameron CH, Briggs M. Virus-like particles in serum of patients with Australia-antigen-associated hepatitis. Lancet 1970; 1:695–8.
16. Hilleman MR, Buynak EB, Roehm RR, Tytell AA, Bertland AU, Lampson GP. Purified and inactivated human hepatitis B vaccine: progress report. Am J Med Sci 1975; 270:401–4.
17. Purcell RH, Gerin JL. Hepatitis B subunit vaccine: a preliminary report of safety and efficacy tests in chimpanzees. Am J Med Sci 1975; 270:395–9.
18. Yuen MF, Chen DS, Dusheiko GM, et al. Hepatitis B virus infection. Nat Rev Dis Primers 2018; 4:18035.
19. Van Damme P, Vesikari T. Pediatric vaccines and vaccinations – Hepatitis B vaccines. Cham, Switzerland: Springer, 2017.
20. Okamoto H, Tsuda F, Sakugawa H, et al. Typing hepatitis B virus by homology in nucleotide sequence: comparison of surface antigen subtypes. J Gen Virol 1988; 69:2575–83.
21. Kao J-H, Chen D-S. HBV genotypes: epidemiology and implications regarding natural history. Current Hepatitis Reports 2006; 5:5–13.
22. Sunbul M. Hepatitis B virus genotypes: global distribution and clinical importance. World J Gastroenterol 2014; 20:5427–34.
23. Gerlich WH. Medical virology of hepatitis B: how it began and where we are now. Virol J 2013; 10:239.
24. Hoofnagle JH, Dusheiko GM, Seeff LB, Jones EA, Waggoner JG, Bales ZB. Seroconversion from hepatitis B e antigen to antibody in chronic type B hepatitis. Ann Intern Med 1981; 94:744–8.
25. Krugman S, Overby LR, Mushahwar IK, Ling CM, Frösner GG, Deinhardt F. Viral hepatitis, type B. Studies on natural history and prevention re-examined. N Engl J Med 1979; 300:101–6.
26. Rémy V, Largeron N, Quilici S, Carroll S. The economic value of vaccination: why prevention is wealth. J Mark Access Health Policy 2015; 3: doi: 10.3402/jmahp.v3.29284.
27. Polaris Observatory Collaborators. Global prevalence, treatment, and prevention of hepatitis B virus infection in 2016: a modelling study. Lancet Gastroenterol Hepatol 2018; 3:383–403.
28. Meireles LC, Marinho RT, Van Damme P. Three decades of hepatitis B control with vaccination. World J Hepatol 2015; 7:2127–32.
29. Francis DP, Feorino PM, McDougal S, et al. The safety of the hepatitis B vaccine. Inactivation of the AIDS virus during routine vaccine manufacture. JAMA 1986; 256:869–72.
30. Shouval D. Hepatitis B vaccines. J Hepatol 2003; 39(suppl 1):S70–6.
31. Emini EA, Ellis RW, Miller WJ, McAleer WJ, Scolnick EM, Gerety RJ. Production and immunological analysis of recombinant hepatitis B vaccine. J Infect 1986; 13 (suppl A):3–9.
32. Stephenne J. Development and production aspects of a recombinant yeast-derived hepatitis B vaccine. Vaccine 1990; 8 (suppl):S69–73; discussion S79–80.
33. Van Den Ende C, Marano C, Van Ahee A, Bunge EM, De Moerlooze L. The immunogenicity and safety of GSK’s recombinant hepatitis B vaccine in adults: a systematic review of 30 years of experience. Expert Rev Vaccines 2017; 16:811–32.
34. Keating GM, Noble S. Recombinant hepatitis B vaccine (Engerix-B): a review of its immunogenicity and protective efficacy against hepatitis B. Drugs 2003; 63:1021–51.
35. Shouval D, Roggendorf H, Roggendorf M. Enhanced immune response to hepatitis B vaccination through immunization with a Pre-S1/Pre-S2/S vaccine. Med Microbiol Immunol 2015; 204:57–68.
36. Kane MA. Global status of hepatitis B immunisation. Lancet 1996; 348:696.
37. Hepatitis B virus: a comprehensive strategy for eliminating transmission in the United States through universal childhood vaccination. Recommendations of the Immunization Practices Advisory Committee (ACIP). MMWR Morb Mortal Wkly Rep 1991; 40(RR-13):1–25.
38. Expanded programme on immunization. Global advisory group--part I. Wkly Epidemiol Rec 1992; 67:11–15.
39. Kao JH, Chen DS. Global control of hepatitis B virus infection. Lancet Infect Dis 2002; 2:395–403.
40. Implementation of hepatitis B birth dose vaccination— worldwide, 2016. Wkly Epidemiol Rec 2018; 93:61–72.
41. Schillie S, Walker T, Veselsky S, et al. Outcomes of infants born to women infected with hepatitis B. Pediatrics 2015; 135:e1141–7.
42. Marion SA, Tomm Pastore M, Pi DW, Mathias RG. Longterm follow-up of hepatitis B vaccine in infants of carrier mothers. Am J Epidemiol 1994; 140:734–46.
43. World Health Organization (WHO). Fact sheet: immunization coverage. Geneva, Switzerland: WHO, 2019.
44. Gentile I, Borgia G. Vertical transmission of hepatitis B virus: challenges and solutions. Int J Womens Health 2014; 6:605–11.
45. World Health Organization (WHO). Implementation of hepatitis B birth dose vaccination—worldwide, 2016. Geneva, Switzerland: WHO, 2018.
46. World Health Organization (WHO). Introduction of hepatitis B vaccine into childhood immunization services. Management guidelines including information for health workers and parents. Geneva, Switzerland: WHO, 2001.
47. Schillie S, Vellozzi C, Reingold A, et al. Prevention of hepatitis B virus infection in the United States: recommendations of the Advisory Committee on Immunization Practices. MMWR Morb Mortal Wkly Rep 2018; 67:1–31.
48. World Health Organization (WHO). Recommendations to assure the quality, safety and efficacy of recombinant hepatitis B vaccines. WHO Technical report series No. 978. Geneva, Switzerland: WHO, 2013.
49. Wiesen E, Diorditsa S, Li X. Progress towards hepatitis B prevention through vaccination in the Western Pacific, 1990–2014. Vaccine 2016; 34:2855–62.
50. Nayagam S, Thursz M, Sicuri E, et al. Requirements for global elimination of hepatitis B: a modelling study. Lancet Infect Dis 2016; 16:1399–408.
51. Ni YH, Chang MH, Jan CF, et al. Continuing decrease in hepatitis B virus infection 30 years after initiation of infant vaccination program in Taiwan. Clin Gastroenterol Hepatol 2016; 14:1324–30.
52. Wenzel JJ, Jilg W. Loss of antibodies, but not of protection. Lancet Infect Dis 2010; 10:738–9.
53. Jack AD, Hall AJ, Maine N, Mendy M, Whittle HC. What level of hepatitis B antibody is protective? J Infect Dis 1999; 179:489–92.
54. Bruce MG, Bruden D, Hurlburt D, et al. Antibody levels and protection after hepatitis B vaccine: results of a 30-year follow-up study and response to a booster dose. J Infect Dis 2016; 214:16–22.
55. Van Damme P. Long-term protection after hepatitis B vaccine. J Infect Dis 2016; 214:1–3.
56. Leuridan E, Van Damme P. Hepatitis B and the need for a booster dose. Clin Infect Dis 2011; 53:68–75.
57. Tabrizi SN, Brotherton JM, Kaldor JM, et al. Assessment of herd immunity and cross-protection after a human papillomavirus vaccination programme in Australia: a repeat cross-sectional study. Lancet Infect Dis 2014; 14:958–66.
58. Beutels P. Economic evaluations of hepatitis B immunization: a global review of recent studies (1994-2000). Health Econ 2001; 10:751–74.
59. La Torre G, Mannocci A, Saulle R, et al. Economic evaluation of HBV vaccination: A systematic review of recent publications (2000–2013). Hum Vaccin Immunother 2016; 12:2299–311.
60. Margolis HS, Coleman PJ, Brown RE, Mast EE, Sheingold SH, Arevalo JA. Prevention of hepatitis B virus transmission by immunization. An economic analysis of current recommendations. JAMA 1995; 274:1201–8.
61. Luyten J, Beutels P. The social value of vaccination programs: beyond cost-effectiveness. Health Aff (Millwood) 2016; 35:212–8.
62. Rodrigues CMC, Plotkin SA. Impact of vaccines; health, economic and social perspectives. Front Microbiol 2020; 11:1526.
63. Van Damme P, Cramm M, Safary A, Vandepapelière P, Meheus A. Heat stability of a recombinant DNA hepatitis B vaccine. Vaccine 1992; 10:366–7.
64. Kolwaite AR, Xeuatvongsa A, Ramirez-Gonzalez A, et al. Hepatitis B vaccine stored outside the cold chain setting: a pilot study in rural Lao PDR. Vaccine 2016; 34:3324–30.
65. Breakwell L, Anga J, Dadari I, Sadr-Azodi N, Ogaoga D, Patel M. Evaluation of storing hepatitis B vaccine outside the cold chain in the Solomon Islands: Identifying opportunities and barriers to implementation. Vaccine 2017; 35:2770–4.
66. Scott N, Palmer A, Morgan C, et al. Cost-effectiveness of the controlled temperature chain for the hepatitis B virus birth dose vaccine in various global settings: a modelling study. Lancet Glob Health 2018; 6:e659–67.
67. Janssen JM, Heyward WL, Martin JT, Janssen RS. Immunogenicity and safety of an investigational hepatitis B vaccine with a Toll-like receptor 9 agonist adjuvant (HBsAg-1018) compared with a licensed hepatitis B vaccine in patients with chronic kidney disease and type 2 diabetes mellitus. Vaccine 2015; 33:833–7.
68. Heyward WL, Kyle M, Blumenau J, et al. Immunogenicity and safety of an investigational hepatitis B vaccine with a Toll-like receptor 9 agonist adjuvant (HBsAg-1018) compared to a licensed hepatitis B vaccine in healthy adults 40-70 years of age. Vaccine 2013; 31:5300–5.
69. A two-dose hepatitis B vaccine for adults (Heplisav-B). Med Lett Drugs Ther 2018; 60:17–8.
70. Janssen RS, Mangoo-Karim R, Pergola PE, et al. Immunogenicity and safety of an investigational hepatitis B vaccine with a toll-like receptor 9 agonist adjuvant (HBsAg-1018) compared with a licensed hepatitis B vaccine in patients with chronic kidney disease. Vaccine 2013; 31:5306–13.
71. Zanella B, Bechini A, Boccalini S, et al. Hepatitis B seroprevalence in the pediatric and adolescent population of Florence (Italy): An update 27 years after the implementation of universal vaccination. Vaccines 2020; 8:156.
72. Bini C, Grazzini M, Chellini M, et al. Is hepatitis B vaccination performed at infant and adolescent age able to provide long-term immunological memory? An observational study on healthcare students and workers in Florence, Italy. Hum Vaccin Immunother 2018; 14:450–5.
73. Osiowy C. From infancy and beyond… ensuring a lifetime of hepatitis B virus (HBV) vaccine-induced immunity. Hum Vaccin Immunother 2018; 14:2093–7.
74. Lee BX, Kjaerulf F, Turner S, et al. Transforming our world: implementing the 2030 agenda through sustainable development goal indicators. J Public Health Policy 2016; 37(suppl 1):13–31.
